# Fatty Acid Binding Protein 4—A Circulating Protein Associated with Peripheral Arterial Disease in Diabetic Patients

**DOI:** 10.3390/jcm9092843

**Published:** 2020-09-02

**Authors:** Abdelrahman Zamzam, Muzammil H. Syed, Elisa Greco, Mark Wheatcroft, Shubha Jain, Hamzah Khan, Krishna K. Singh, Thomas L. Forbes, Ori Rotstein, Rawand Abdin, Mohammad Qadura

**Affiliations:** 1 Division of Vascular Surgery, St. Michael’s Hospital, Toronto, ON M5B 1W8, Canada; abdelrahman.zamzam@unityhealth.to (A.Z.); muzammil.syed@mail.utoronto.ca (M.H.S.); elisa.greco@unityhealth.to (E.G.); mark.wheatcroft@unityhealth.to (M.W.); jains@ucalgary.ca (S.J.); hamzah.khan@mail.utoronto.ca (H.K.); 2 Department of Surgery, University of Toronto, Toronto, ON M5S 1A1, Canada; thomas.forbes@uhn.ca (T.L.F.); ori.rotstein@unityhealth.to (O.R.); 3 Department of Medical Biophysics, Schulich School of Medicine and Dentistry, University of Western Ontario, London, ON N6A 5C1, Canada; krishna.singh@uwo.ca; 4 Division of Vascular Surgery, Peter Munk Cardiac Centre, University Health Network, University of Toronto, Toronto, ON M5G 2N2, Canada; 5 Keenan Research Centre for Biomedical Science, Li Ka Shing Knowledge Institute of St. Michael’s Hospital, Toronto, ON M5B 1W8, Canada; 6 Department of Medicine, McMaster University, Hamilton, ON L8S 4K1, Canada; rawand.abdin@medportal.ca

**Keywords:** diabetes mellitus, peripheral arterial disease, biomarker, protein, correlation

## Abstract

Diabetic patients with peripheral arterial disease (PAD) often suffer from poor clinical outcomes such as limb-loss. Fatty acid binding protein 4 (FABP4) is mainly expressed by adipocytes and is known to play a significant role in the development of atherosclerosis. In this study, we sought to investigate whether FABP4 is associated with PAD in patients with type 2 diabetes mellitus (DM). FABP4 plasma levels were studied in 119 diabetic patients with PAD (DM-PAD) and 49 diabetic patients without PAD (DM-noPAD) presenting to St. Michael’s Hospital between October 2017 and September 2018. Levels of FABP4 in DM-PAD patients (23.34 ± 15.27 ng/mL) were found to be over two-fold higher than the levels in DM-noPAD patients (10.3 ± 7.59 ng/mL). Regression analysis demonstrated a significant association between FABP4 levels and DM-PAD after adjusting for age, sex, prior history of coronary arterial disease and white blood cells count (OR, 2.77; 95% CI, 1.81–4.31; *p*-value = 0.001). Relative to DM-noPAD controls, plasma FABP4 levels in DM-PAD patients were noted to be inversely correlated with the ankle brachial index (ABI; r= −0.374, *p*-value < 0.001). The diagnostic ability of FABP4 was investigated using receiver operator curves (ROC) and area under the curve (AUC) analysis. FABP4 had an AUC of 0.79, which improved to 0.86 after adjusting for age, sex and prior history of coronary arterial disease. This raises a possibility of utilizing FABP4 as a biomarker for diagnosing PAD in diabetic patients.

## 1. Introduction

Type 2 diabetes mellitus (DM) is a serious chronic condition that affects over 400 million patients globally [1]. Patients with DM are at an increased risk of developing various cardiovascular diseases, such as peripheral arterial disease (PAD) [2]. PAD is caused by the development of atherosclerotic plaques within the arterial vessels supplying blood to the limbs, resulting in lower limb claudication, rest pain or even tissue loss (in symptomatic patients). Despite the best medical therapy, PAD patients are still at higher risk of all-cause mortality when compared to patients without PAD [3]. Moreover, patients suffering from both DM and PAD concurrently are at a higher risk of having cardiovascular events, lower limb amputations, and death [4,5,6].

The pathophysiology by which DM causes changes in lower limb vasculature and PAD is complex. Studies have proposed that chronic glycemic stress, chronic low-grade inflammation, and impaired vascular tissue repair are a few of the pathways that may be involved in the development of PAD [7,8].

Fatty acid-binding proteins (FABPs) belong to a multigene family encoding approximately 15-kDa proteins [9]. These proteins traffic hydrophobic ligands throughout cellular compartments [9,10]. Furthermore, each protein belonging to this family has unique tissue-specific expression patterns that differentiate it from other members of the family [11]. Adipocytes and macrophages are known to produce fatty acid binding protein 4 (FABP4) [12,13]. This protein is usually released into circulation from adipocytes during lipolysis and possibly acts as an adipokine [14].

Several studies have demonstrated that FABP4 is associated with obesity [15], as well as insulin resistance and metabolic syndrome [16]. However, no previous study has examined the possible association between FABP4 and PAD in patients with diabetes. Therefore, we sought to assess whether circulating FABP4 is independently associated with PAD in patients with diabetes, and its potential to serve as a diagnostic biomarker in this regard.

## 2. Experimental Section

### 2.1. Ethics Approval

This study was approved by The Unity Health Toronto Research Ethics Board at St. Michael’s Hospital–University of Toronto in Ontario, Canada (#16-375, 8 February 2017). Consent was obtained from all patients.

### 2.2. Patient Selection

In this pilot study, we prospectively evaluated consecutive diabetic patients with PAD (DM-PAD) and diabetic patients without PAD (DM-noPAD) presenting to ambulatory vascular surgery clinics at St. Michael’s Hospital between October 2017 and September 2018. The DM-noPAD patients were defined as patients with a normal arterial ultrasound (US) of the lower limbs, ankle brachial index (ABI) of ≥0.9 and/or toe brachial index (TBI) of ≥0.67, palpable posterior tibial and dorsalis pulses in each leg, and no clinical history of claudication. In contrast, the DM-PAD patients were defined as ABI <0.9 or TBI <0.67 and had a lack of posterior tibial and dorsalis pulses in at least one leg, with or without claudication.

The exclusion criteria for the study were: (1) patients without DM, (2) patients with type 1 diabetes, (3) patients on chemotherapy, (4) patients with renal disease (stages 3, 4 and 5) as per the Kidney Disease Outcomes Quality Initiative clinical guidelines and, (5) patients with acute limb ischemia, elevated troponins, or a 3-month history of acute coronary syndrome (ACS), as defined by the American College of Cardiology.

### 2.3. Baseline Measurements

Type 2 DM was defined as glycosylated hemoglobin A1c ≥6.5% or the use of anti-diabetic medication. Hyperlipidemia was defined as total cholesterol >5.2 mmol/L, triglyceride >1.7 mmol/L, or the use of anti-hyperlipidemic medication. Hypertension was defined as systolic blood pressure ≥130mm Hg, diastolic pressure ≥80 mm Hg, or the use of antihypertensive medication. Renal disease was defined as estimated glomerular filtration rate less than 59 mL/min/1.73 m^2^, as per the Kidney Disease Outcomes Quality Initiative 2002 guidelines. Prior history of coronary arterial disease, stroke, or transient ischemic attack (TIA) was recorded for each patient, in addition to smoking status.

### 2.4. PAD Assessment and Sample Processing

A thorough physical exam was conducted, and a complete medical history was obtained from each patient. Each patient also underwent a lower limb arterial US including ABI or TBI, which was documented and recorded. Blood samples were drawn into vacutainer tubes containing EDTA. Plasma was then extracted from this blood via centrifugation at 3000 rpm for 10 min at 4 °C.

### 2.5. FABP4 Multiplex Assay

Plasma samples were analyzed in duplicate using MILLIPLEX MAP Human Cardiovascular Disease (CVD) Magnetic Bead Panel 1 (EMD-Millipore; Billerica, MA, USA) to determine the concentrations of FABP4. All sample analyses were completed on the same day to eliminate inter-assay variability. Sample intra-assay and inter-assay CV were both <10%. The MagPix analyzer (Luminex Corp; Austin, TX, USA) was calibrated prior to analysis using Fluidics Verification and Calibration bead kits (Luminex Corp). A minimum of 50 beads for each targeted biomarker were acquired using Luminex xPonent software and analyzed using Milliplex Analyst software (v.5.1; EMD-Millipore, Darmstadt, Germany.).

### 2.6. Statistical Methods

Demographics and clinical characteristics were expressed as means with standard deviations or percentages (%). Continuous data for DM-PAD and DM-noPAD groups were compared using Student’s *t*-test if the data were normally distributed, otherwise the Mann–Whitney U test was used. Chi-square test was used for comparing categorical variables in the DM-PAD and DM-noPAD groups. Correlations between FABP4 and ABI were analyzed using Spearman’s correlation. Probit regression was used to calculate the odds ratios (ORs) with 95% confidence intervals (95% CI) for the development of PAD given FABP4 plasma concentration levels of patients. Standardized FABP4 values were used for ease of odds ratio interpretation. Multiple probit regression analyses were used to examine the association of FABP4 and other parameters with the development of PAD. Area under the curve (AUC) from receiver operating characteristic (ROC) analysis was used to determine how well FABP4 can distinguish between DM-PAD and DM-noPAD groups. All analyses were carried out at a 5% two-sided significance level and carried out using SPSS software version 23 (SPSS Inc., Chicago, IL, USA).

## 3. Results

### 3.1. Cohort Description

168 patients with diabetes met our study criteria, of which 119 had PAD (DM-PAD) and 49 did not (DM-noPAD). Mean age of the patients was 69 years, with 79% of the cohort comprised of males. Within the DM-PAD patients, there were 17 patients (10%) with ischemic ulcers while none of the DM-noPAD had any ulceration. With the exception of ABI, prior history of coronary arterial disease (CAD), white blood cells count (WBC), and ulceration, there were no other significant differences found between both groups (Table 1). Furthermore, the circulating levels of FABP4 in the DM-PAD group were found to be approximately two-fold higher than the DM-noPAD group (23.34 ± 15.27 ng/mL vs. 10.30 ± 7.59 ng/mL, respectively; *p*-value = 0.001), even after removing patients with ulceration, (23.12 ± 15.77 ng/mL for DM-PAD vs. 10.30 ± 7.59 ng/mL for DM-noPAD; *p*-value = 0.001).

### 3.2. Regression Analysis

To investigate the influence of confounding factors on FABP4 levels, multiple stepwise regression analyses were conducted on the 119 DM-PAD and the 49 DM-noPAD patients (Table 2). We found that FABP4 levels were independently and strongly associated with DM-PAD status, despite adjusting for age and sex (model 1) (OR, 2.74; 95% CI, 1.80–4.18; *p*-value = 0.001). Table 2 highlights additional models that demonstrate a significant association between FABP4 levels and PAD after adjusting for other factors. In the final model, after adjusting for all tested variables, a significant likelihood of having PAD per one standard deviation increase in FABP4 levels (OR, 2.77; 95% CI, 1.81–4.31; *p*-value = 0.001) was observed. Furthermore, sensitivity analysis was conducted for the final model results, in which regression analysis was conducted after removing the 17 patients with ulceration in the DM-PAD group. The estimated odds ratio for the sensitivity analysis was found to be 2.73 (95% CI, 1.79–4.22; *p*-value = 0.001).

### 3.3. Hemodynamic Association between FABP4 and DM-PAD

**Figure 1:**
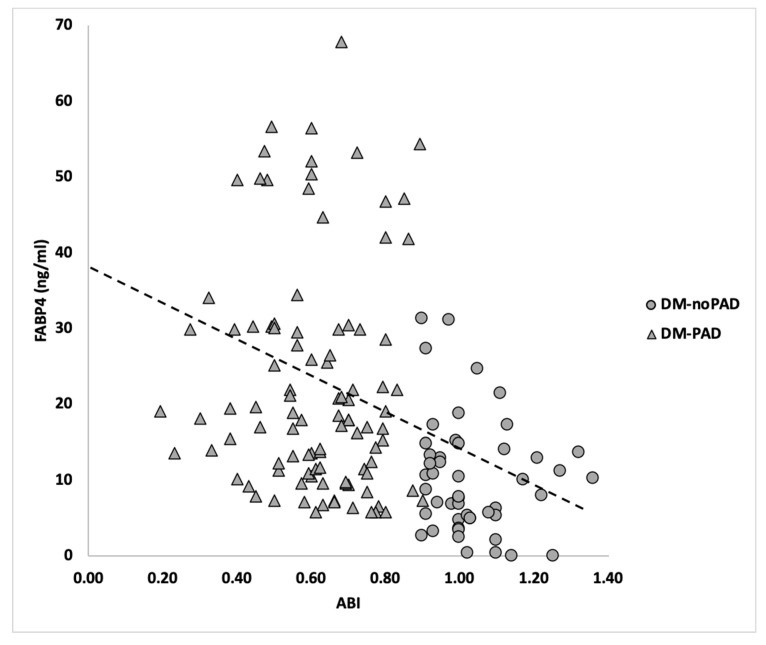
**Relationship between FABP4 and DM-PAD.** Spearman correlation between ankle brachial index (ABI) and fatty acid binding protein 4 (FABP4) in DM-noPAD (diabetic patients without peripheral arterial disease, *n* = 49, circles) and DM-PAD (diabetic patients with peripheral arterial disease, *n* = 119, triangles). FABP4 values were inversely correlated to the ABI using spearmen correlation (r = −0.374, *p*-value = 0.001).

We studied the hemodynamic correlation between FABP4 and ABI to better understand the association between FABP4 and DM-PAD. Relative to the DM-noPAD patients, plasma FABP4 levels of DM-PAD subjects were inversely correlated with ABI (r = −0.374, *p*-value <0.001; Figure 1)

### 3.4. Diagnostic Potential of FABP4 for PAD within Diabetic Patients

Finally, ROC analysis was performed to measure the diagnostic accuracy of FABP4. Prior to any adjusting, an AUC of 0.79 with a sensitivity of 76.4% and specificity of 65.7% was observed. After adjusting for age, sex and prior history of coronary arterial disease, the ROC analysis demonstrated an improved AUC of 0.84 with a sensitivity of 80.2% and specificity of 79.7%.

## 4. Discussion

In this study, we demonstrated that FABP4 levels are significantly elevated in diabetic patients with PAD, even after accounting for potential confounding factors (as compared to diabetic patients without PAD). The robustness of this finding was also examined through a sensitivity analysis and still held true. Furthermore, correlation studies demonstrated that as PAD status worsens hemodynamically within diabetic patients, circulating levels of FABP4 tend to increase. These data suggest that FABP4 levels appear to escalate as the severity of PAD increases in diabetic patients. Therefore, these data demonstrate an independent association between FABP4 and PAD in patients with diabetes.

Fatty acid binding protein 4 (FABP4), also known as adipose fatty acid binding protein (AFABP), is a low-molecular weight protein that is abundantly expressed in mature adipocytes [14], and has previously been shown to be associated with several metabolic disease states such as obesity, metabolic syndrome, type 2 diabetes, and insulin resistance [17,18]. Previously, it has been reported that circulating FABP4 may be a potential biomarker for the progression of nephropathy in type 2 diabetic patients [19]. Furthermore, several studies have demonstrated that circulating FABP4 may also predict the development of atherosclerotic diseases [20]. For instance, Chow et al. suggested that circulating FABP4 levels predict the development of cardiovascular disease—defined as acute myocardial infarction, angina pectoris, stroke, and heart failure [21].

As a result, FABP4 has been investigated as a potential biomarker of atherosclerosis. Miysohi et al. investigated the association between the FABP4 levels and coronary atherosclerosis in 125 patients, and concluded that circulating FABP4 levels could be utilized for the evaluation of the extent of coronary atherosclerosis [22]. Similarly, Rhee et al. demonstrated that circulating FABP4 levels increased within Korean adults as the numbers of stenotic coronary artery increased, but these differences were curtailed after adjusting for confounding factors such as age and fasting glucose levels [23]. Likewise, increased serum FABP4 has also been shown to be significantly associated with a greater coronary plaque burden [24].

However, only a limited number of studies have previously investigated the association between FABP4 and PAD. For instance, Hobaus et al. demonstrated a strong association between elevated FABP4 levels and future cardiovascular events over 5 years in 327 PAD patients. The authors also showed that elevated FABP4 levels were associated with death, nonlethal myocardial infarction, and nonfatal stroke. These data favored the proposition of utilizing circulating FABP4 as a biomarker for future cardiovascular events in patients with PAD [25]. In contrast, we demonstrated (for the first time) that FABP4 is associated with PAD as well as PAD severity within diabetic patients. Moreover, we also noted an AUC of 0.84 for FABP4 in PAD patients after adjusting for confounding factors. This suggests that FABP4 can potentially serve as a biomarker for PAD within diabetic patients. Although larger studies are needed to confirm and validate these findings, this study builds a strong foundation to design clinical trials in this regard. Furthermore, while investigating the mechanisms behind our findings are beyond the scope of this paper, we believe that the increased atherosclerotic burden in patients with advanced PAD may explain the increasing trend of FABP4 levels with worsening PAD severity as shown in Figure 1. This is in accordance with previous studies that have demonstrated altered protein profiles for advanced PAD patients as compared to healthy controls or patients with mild/moderate PAD [26,27,28]. Alternatively, other etiologies such as increased ischemia may influence the release of adipokines from adipocytes.

Although this is the first study that sought to investigate the association between FABP4 and diabetic patients with PAD, it is not without limitations. First, our study is a small, single-center study and thus the findings of this study may not be generalizable. Second, we cannot rule out residual or unmeasured confounding factors as an alternative explanation of our results.

Conversely, this study also has strengths that we feel warrant attention. For instance, to the best of our knowledge, no other study has previously investigated the association between FABP4 and PAD within diabetic patients, nor the diagnostic potential of this protein in identifying PAD within diabetic patients. Furthermore, the statistical analysis used in this study can be considered robust.

## 5. Conclusions

In this study, we demonstrated that circulating levels of FABP4 are elevated in PAD patients with diabetes. We also noted a positive correlation between increasing FABP4 levels and increasing PAD severity in diabetic patients, even after adjusting for potential confounding factors. Therefore, our data support the role of FABP4 as a possible biomarker for PAD in diabetic patients.

## Figures and Tables

**Table 1:** Patient demographics and clinical characteristics.

Variable	Non-PAD (*n* = 49)	PAD (*n* = 119)	*p*-Value ^α^
Mean (SD) ^†^			
ABI	1.03 (0.11)	0.61 (0.15)	0.001
Age in years	68.3 (12.8)	69.1 (9.29)	0.628
Frequency (%) ^‡^			
Sex-male *n* (%)	38 (79)	82 (70)	0.159
Hypertension *n* (%)	38 (79)	101 (86)	0.214
Hypercholesteremia *n* (%)	38 (79)	98 (83)	0.350
Renal Insufficiency *n* (%)	6 (13)	13 (11)	0.789
Smoking *n* (%)	35 (76)	90 (78)	0.494
CAD *n* (%)	15 (32)	63 (53)	0.016
Stroke *n* (%)	7 (21)	21 (18)	0.803
Ulceration *n* (%)	0 (0)	17 (10)	0.001
ASA *n* (%)	28 (57)	76 (64)	0.485
ACEi/Arb *n* (%)	19 (56)	78 (71)	0.142
Beta-Blocker *n* (%)	9 (27)	48 (43)	0.108
CCB *n* (%)	9 (27)	33 (30)	0.830
Insulin *n* (%)	7 (21)	25 (23)	0.820
Oral Hypoglycemia *n* (%)	18 (55)	76 (69)	0.145
Mean Blood Work mean (SD)			
Cr (mmol/L)	87.0 (29.9)	92.8 (54.7)	0.934
INR	1.39 (0.95)	1.15 (0.26)	0.148
WBC (×10^9^)	8.30 (2.09)	9.32 (2.50)	0.033
Platelet count (×10^3^)	213 (64.1)	230 (66.5)	0.179
HbA1c (%)	0.07 (0.02)	0.07 (0.01)	0.409
HDL (mmol/L)	0.99 (0.23)	0.99 (0.25)	0.815
LDL (mmol/L)	1.49 (0.37)	1.76 (0.80)	0.143
Glucose (mmol/L)	8.3 (2.49)	7.9 (2.59)	0.197
Triglycerides (mmol/L)	2.03 (1.64)	2.35 (3.09)	0.893

ABI: ankle brachial index; ASA: aspirin; ACEi/ARB: angiotensin-converting-enzyme inhibitors/angiotensin II receptor blocker; CAD: coronary arterial disease; CCB: calcium channel blockers; Cr: creatinine; WBC: white blood cells count; HbA1c: hemoglobin A1c; INR: international normalized ratio; LDL: low-density lipoprotein; HDL High-density lipoprotein; Non-PAD: no peripheral arterial disease; PAD: peripheral arterial disease; ^α^ the significance of the difference between DM-PAD (diabetic patients with peripheral arterial disease) and DM-noPAD (diabetic patients without peripheral arterial disease) groups; ^†^ differences between groups were compared using Mann–Whitney test; ^‡^ differences between groups were compared using chi-square test; means and standard deviations were calculated for continuous variables; all numbers were rounded to one decimal place; frequencies and percentages were calculated for categorical variables; all numbers were rounded up with zero decimal place; all *p*-values were rounded to three decimal places.

**Table 2:** Influence of individual factors on the odds ratios for DM-PAD per one standard deviation increase in plasma fatty acid-binding protein 4.

Regression Models	Odds Ratio (95% CI) ^‡^	*p*-Value
**Model 1,** adjusted for age and sex	2.74 (1.80–4.18)	0.001
**Model 1 + CAD**	2.76 (1.79–4.29)	0.001
**Model 1 + CAD + WBC**	2.77 (1.81–4.31)	0.001

Model 1: DM-PAD (no/yes) as the dependent variable; CAD: coronary arterial disease; WBC: white blood cells count; ^‡^ probit regression models for peripheral arterial disease (PAD) per one standard deviation increase in fatty acid binding protein 4 (FABP4) levels (ng/mL).
